# Rapid Breathing as a Pain Obtunder

**Published:** 1880-10

**Authors:** 


### ARTICLE VI.

### *Rapid Breathing as a Pain Obtunder.*

In 1875 Dr. W. G. A. Bonwill a dentist of Philadelphia, discovered that by causing his patients to breathe rapidly for a few minutes, the sense of pain was often so obtunded that he could extract teeth without causing any discomfort. The matter was, soon after this, taken up by Dr. Addinell Hewson, who made a favorable report of his experience with the method, at the International Medical Congress in 1876. Not much interest was excited, however, and the subject was virtually dropped.

At a recent meeting of the Philadelphia County Medical Society, papers upon rapid breathing, as a means of in ducing analgesia, were read by Dr. Benjamine Lee and by Dr. Bonwill. Considerable discussion followed. A perusal of the papers and discussion leads to the conclusion that there may be something of considerable practical value in Dr. Bonwill's discovery. At any rate, as it is a thing which can be easily tried without risk to any one, we give briefly the facts regarding it.

In some cases it is necessary, when rapid breathing is to be undertaken, to have the patient sitting in a chair. The most favorable position for him, however, is that of lying down on the side; and it is generally best to throw a handkerchief over the face, so as to prevent the patient's attention from being distracted. He should then be made to breath at the rate of about one hundred respirations per minute. The direction best given is to "blow out" in rapid puffing expirations. At the end of from two to five minutes, the patient continuing his rapid breathing all the time, teeth may be drawn or incisions made, and there will generally be an entire or partial absence of pain, which will last thirty seconds or more. The sense of touch is not affected, nor is unconsciousness gone. When the breathing is first begun the patient may feel some exhilaration; following this comes a sensation of fulness in the head or dizziness. The face during this time becomes at first flushed, but later, according to Dr. Hewson, it is pale or even bluish. The heart beats more feebly and somewhat faster than normal. We are told that this phenomenon of analgesia is produced in females more readily than in males, and in those of middle age more easily than in the young or old. Children under ten can rarely be made to breathe properly. In cold, clear air a longer time is required than when it is warm.

Regarding the practical uses of the method, it is claimed that it may supplant ether, chloroform, or nitrous oxide in dentistry, minor surgery, and often in obstetrics. In the latter case it is especially applicable when the forceps are to be used. A case of tracheotomy and one of ischio-rectral abscess were related, in which analgesia was successfully produced by rapid breathing. The ordinary anæsthetics, when employed for major operations, can be made more effective by combining with them rapid breathing. According to Drs. Garretson, Hewson and Kite, it takes from one half to three-fourths less of the liquid anæsthetics to produce insensibility when the administration is supplemented by rapid breathing. The after effects are less nauseating and unpleasant.

As regards the possible dangers, it is denied that there can be any. Dr. W. R. D. Blackwood, however, stated that he had tried the method on a somewhat hysterical patient, who kept on breathing rapidly for so long a time that he was somewhat alarmed.

The theories of how the rapid breathing acts in thus obtunding pain are as yet unsatisfactory. Dr. Bonwill and Dr. Hewson, its two especial champions, entirely disagree in their attempts at explanation. Dr. Bonwell assigns three causes: 1st, the division of the will-force caused by the rapid voluntary muscular action; 2d, the specific effect of carbonic acid gas set free from the tissues, caused by the throwing into the lungs five times the normal amount of oxygen; 3rd, hyperæmia, due to the excessive amount of air passing into the lungs and causing a damming up of the blood in the brain. The main point of the theory is that rapid breathing produces a hyperoxidation of the blood, which sets carbonic acid free in the tissues. Dr. Hewson, on the other hand, believes that there is a diminished oxygenation of the blood during rapid-breathing, and that the excess of carbonic acid which results poisons the nerve-centres and causes analgesia.

It is unnecessary to discuss these theories. The one is crude, and neither is sufficient. As far as the method itself is concerned, it seems to be well proved that analgesia to a greater or less extent can be obtained by it. Its practical usefulness is not so well established. A good many failures have occurred, and a large amount of evidence is yet to be obtained in order to show its reliability. The method is simple, however, and there ought not to be any trouble in speedy determining its exact value.—*Med. Record.*

---

